# Asymptomatic Wolff-Parkinson-White with shortest pre-excited R-R interval during atrial fibrillation ≤250 ms: When to go invasive?

**DOI:** 10.1016/j.hrcr.2024.06.005

**Published:** 2024-06-14

**Authors:** Csaba Herczku, Lennart Bergfeldt

**Affiliations:** ∗ Department of Molecular and Clinical Medicine, Institute of Medicine, Sahlgrenska Academy, University of Gothenburg, Gothenburg, Sweden; † Region Västra Götaland, Department of Cardiology, Sahlgrenska University Hospital, Gothenburg, Sweden

**Keywords:** Asymptomatic pre-excitation, WPW, Preparticipation health screening, Risk assessment, Atrial fibrillation, Sudden cardiac death

**Key Teaching Points**

• Asymptomatic pre-excitation does not exclude the presence of a high-risk accessory pathway, as this case shows.
• Even though the a priori risk for sudden cardiac death is low in patients with asymptomatic pre-excitation, the risk adds up over time. There is no noninvasive test that completely rules out the presence of a high-risk pathway, and invasive risk assessment including isoprenaline is therefore recommended.
• Ablation of an accessory pathway is presently the only therapy that can abolish a risk factor for sudden cardiac death, with a high probability of success at low risk for complications.
• Surface electrocardiogram (ECG) is helpful to narrow down the proportion of accessory pathways where the risk for high-degree atrioventricular block and other complications might exceed 1%.
• Preparticipation screening with versus without ECG increases the sensitivity of detecting cardiovascular abnormalities associated with sudden cardiac death, including pre-excitation, up to 6 times, and comes at a reasonable cost—but at a price, with false-positives that must be dealt with.

## Introduction

This clinical case is associated with guidelines and statements regarding how to manage asymptomatic preexcitation (aka asymptomatic WPW; Wolff-Parkinson-White) as a special case of impending supraventricular tachycardia and risk for sudden cardiac death (SCD); the role of the 12-lead electrocardiogram (ECG) in screening for cardiovascular disease in young people, athletes as well as non-athletes; and activation-induced cardiac memory.^1^, ^2^, ^3^, ^4^, ^5^

## Case report

In the autumn of 2020, and as part of preparticipation health screening, a 12-lead ECG was taken in a 20-year-old Swedish man invited to play ice hockey on a team of the North American Hockey League (NAHL). Asymptomatic pre-excitation was diagnosed, and a successful ablation of the congenital accessory pathway was required for playing on the team. Because he had never had any symptoms, there was no emergency indication for ablation. Consequently, his Swedish insurance company declined to pay for having the ablation performed locally at an estimated cost of US$40,000. His future was in limbo and his mother called one of the authors, and subsequently the local cardiologist, who referred him to the authors’ hospital for ablation. The patient’s past medical history was unremarkable, the diagnosis and indication for therapy was clear, and he was accepted for an electrophysiology study (EPS) study including ablation.

**Figure 1:**
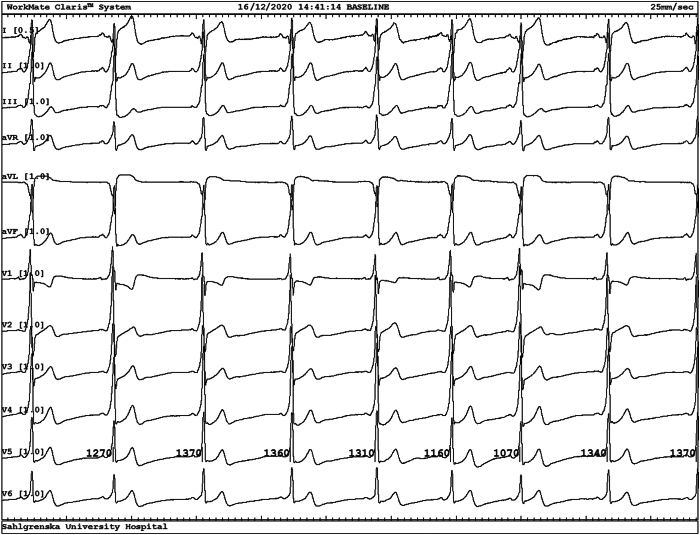
Ventricular pre-excitation during sinus rhythm. Sinus rhythm with ventricular pre-excitation via a left lateral accessory pathway. Recording speed 25 mm/s.

At hospital admission in December 2020, physical examination, ECG, echocardiogram, and routine laboratory tests were performed. Apart from pre-excitation on the ECG, pointing to a left lateral accessory pathway, all tests were completely normal (Figure 1).

**Figure 2:**
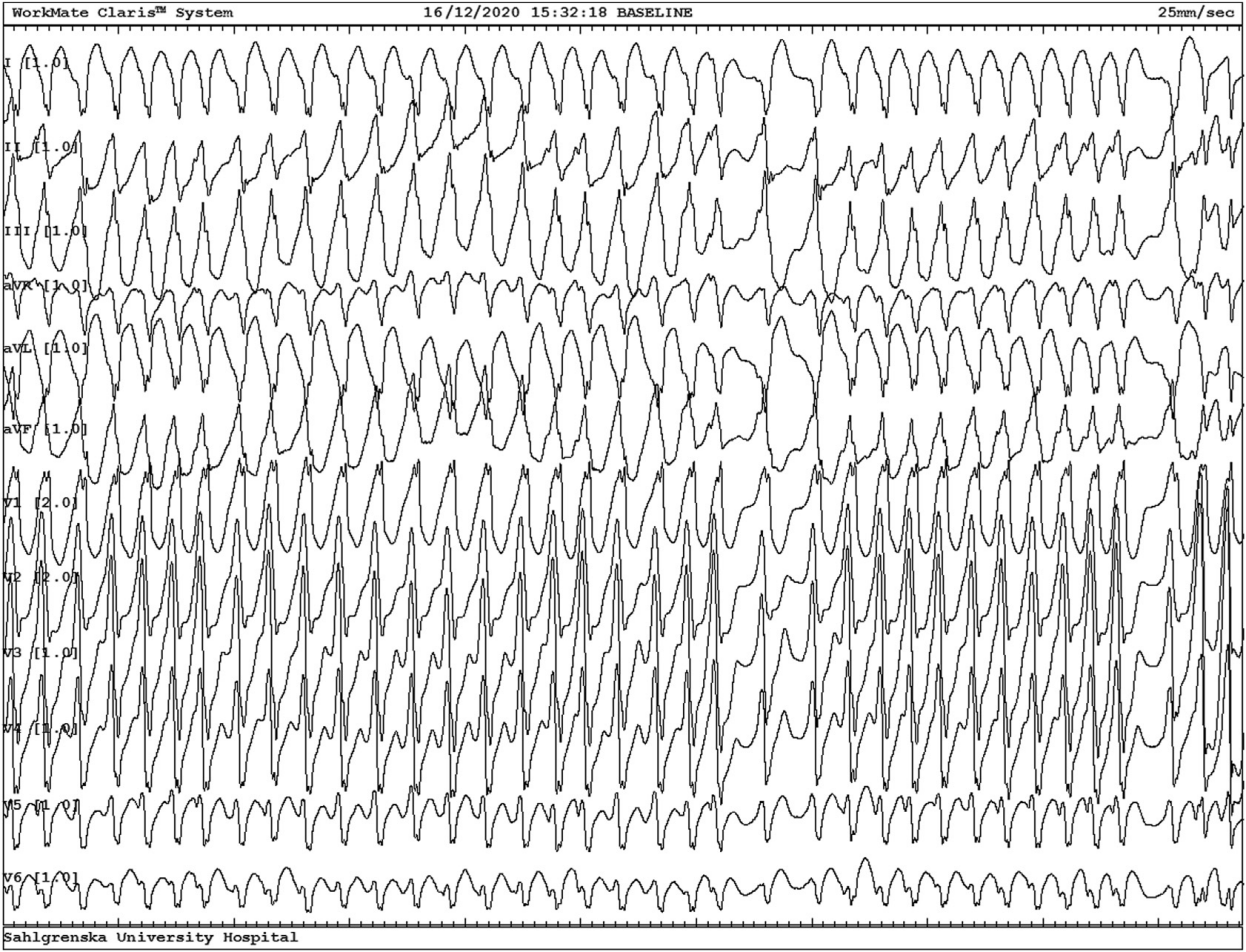
Ventricular pre-excitation during atrial fibrillation. Completely irregular broad QRS complex tachycardia with QRS morphology as during sinus rhythm in Figure 1, ie, pre-excited atrial fibrillation with very short pre-excited R-R intervals suggesting life-threatening properties of the accessory pathway. The patient was conscious but hemodynamically compromised. Recording speed 25 mm/s.

**Figure 3:**
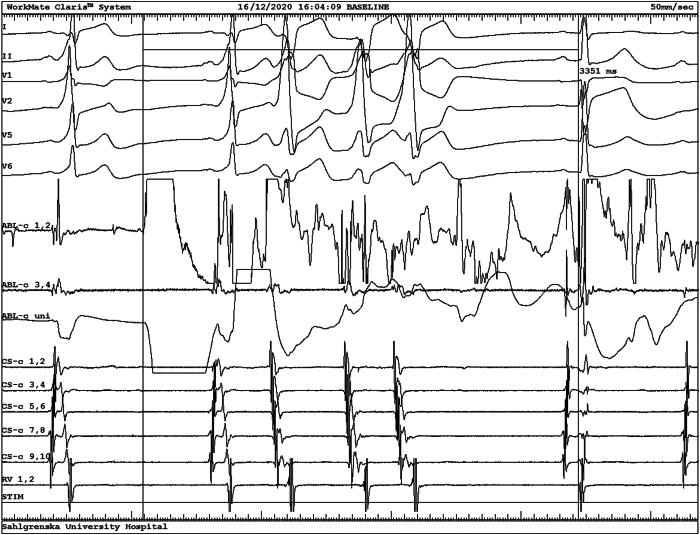
Electrocardiograms at successful radiofrequency (RF) application. Selected electrocardiogram leads are shown above, and below are intracardiac recordings from the ablation catheter in the left atrium (ABL-c1,2, ABL-c3,4, ABL-c uni), from the coronary sinus catheter (CS-c 1,2, … CS-c9,10), and from the right ventricular apex catheter (RV 1,2); numbering starts at the catheter tip. RF application starts at the vertical line with square-wave in ABL-c 1,2. The first cardiac cycle after RF onset is a pre-excited sinus beat. Note the merged A and V in ABL-c 1,2 and the tiny A followed by a very rapid, negative drop in the V in ABL-c uni. The following 3 cycles represent ectopies from the pathway (extremely tight AV signal) and the last cycle sinus rhythm without pre-excitation 3.35 seconds after RF start. Note the ST-T elevation in V_1_–V_2_ in the last beat reflecting postablation cardiac memory. Recording speed 50 mm/s.

According to our routine protocol, the EPS started with ventricular pacing for assessment of the retrograde conduction properties and location of the atrial insertion of the accessory pathway, in our experience the least likely test to initiate an arrhythmia. However, atrial fibrillation (AF) with very rapid heart rate ensued, with low blood pressure and severe discomfort. The shortest pre-excited R-R interval (SPRRI) during AF was 223 ms, which, in addition to the symptoms, showed an accessory pathway with potentially life-threatening properties (Figure 2). The arrhythmia stopped spontaneously, and ablation of a left lateral accessory pathway was successfully performed (Figure 3). Repeated remote follow-up contacts confirmed long-term results and the patient resumed playing ice hockey in the United States.

## Discussion

First, this case concerns the clinical issue of how to manage asymptomatic pre-excitation on routine ECG recorded for whatever reason. This topic was addressed in an American review,^1^ in a European guideline,^2^ and as part of the *Education* series in *Heart*.^3^ Although there are certain signs agreed upon as pointing out high-risk accessory pathways, the predictive value is limited. Furthermore, and as discussed below, there is unfortunately no noninvasive test that rules out future risk for tachycardia (with unpredictable symptoms) or the presence of a high-risk pathway regarding SCD even in patients with asymptomatic pre-excitation in whom the risk might be perceived or even suggested to be low.^3^ A general principle in science is that there is no way to prove that something is true; it might be true if the theory/concept/opinion is not falsified, ie, proven wrong. This case illustrates that asymptomatic pre-excitation does not always mean low risk for a life-threatening event. The notion that asymptomatic WPW is a low-risk feature is thus not applicable when we face an individual patient. So can we ever make an informed decision without an invasive EPS?

Noninvasive risk assessment of accessory pathway properties was not performed in our case because ablation was needed. Holter monitoring and exercise stress test (EST) are the main methods for noninvasive risk assessment of accessory pathway properties, although its sensitivity to pharmacological tests with sodium channel blockers also has been applied. When AF occurs during ECG recording including Holter, an SPRRI ≤250 ms clearly indicates a high-risk accessory pathway, while an SPRRI >250 ms does not rule it out. Intermittent loss of pre-excitation indicates a higher likelihood of the presence of a low-risk but does not rule out a high-risk accessory pathway. EST has been used in several studies and recently in a cohort of 164 consecutive patients with pre-excitation referred for risk assessment (61 asymptomatic); they also underwent invasive EPS with or without isoprenaline.^6^ The main results were as follows: there was no between-group difference in accessory pathway effective refractory period (APERP); APERP and/or SPRRI ≤250 ms was equally common in asymptomatic and symptomatic patients (40% vs 41%; nonsignificant), as were inducible supraventricular arrythmias (61% vs 69%; nonsignificant). The authors' conclusion was that “sudden loss of pre-excitation during EST has a low sensitivity and a low negative predictive value to rule out potentially dangerous APs.”^6^ These results, together with the results of other studies referred to in their discussion, show that a substantial proportion of patients with a diagnosis of asymptomatic WPW during an invasive EPS end up in the category of those with inducible supraventricular tachycardia for which the consensus is that the risk for SCD increases, and ablation should be recommended.^1^, ^2^, ^3^

We agree with Antiperovitch and colleagues^3^ that the midseptal pathways carry the highest risk for inadvertent high-degree atrioventricular (AV) block. To their pertinent discussion about the potential risk-benefit of an EPS ± ablation, we would, however, like to add a few points. In a 2-center study of 1853 patients (15% children) with presumably manifest pre-excitation, right midseptal pathways were found in 4.0% (95% confidence interval 3.2%–5.0%).^7^ Left free wall and posteroseptal pathways made up 84% (51% and 33%, respectively), and they are usually rather easily identified on the surface ECG. Right anteroseptal and right free wall pathways constituted 12% (7% plus 5%) and, like the midseptal pathways, usually have left bundle branch block pattern. Even though the frontal plane axis often is helpful for suggesting where the right-sided pathways are located, the position of the heart in the chest and the rotation along its base-to-apex axis are confounders. Only with catheters inside the heart can the location be decided. So, based on the surface ECG and probability, the proportion of high-risk pathways where the risk for serious complications might exceed 1% can be narrowed down when discussing management options with the patient.

When it comes to the risk assessment for SCD, we favor inclusion of isoproterenol but only as part of the EPS when assessing antegrade accessory pathway conduction properties and when there are no high-risk signs at baseline. A high-risk pathway is thus considered present when the antegrade APERP or SPRRI at baseline is ≤250 ms. When that is not the case, the argument for including isoproterenol, albeit prognostic data are unreliable, is to define the worst-case scenario with regard to antegrade AP conduction properties. In addition, isoproterenol (or other catecholamines) also facilitates tachycardia induction, which is another main goal both for invasive risk assessment and in routine ablation procedures.^6^

When the location is right midseptal, pathway properties seem benign, and we cannot induce tachycardia, we would not attempt ablation prior to another discussion with the patient outside the lab; thus, we would stop the procedure at this point. On the other hand, in the presence of a high-risk accessory pathway or when tachycardia is induced, we would attempt cryoablation. Anteroseptal pathways are also considered to carry an increased risk for inadvertent AV block. We apply several means to minimize this risk: (1) using long sheaths from the groin to stabilize the catheter; (2) to avoid bumping the AP, we perform careful mapping of the tricuspid ring starting in a posteroseptal location going both anterior and lateral, respectively, before approaching the suspected target area; (3) mapping of the atrial insertion during tachycardia when feasible; (4) transiently inhibit pre-excitation by gentle acupressure (clockwise torque of the mapping catheter from the groin), thereby confirming the atrial accessory pathway location; and (5) during radiofrequency ablation, gradually increasing the output. The His bundle itself is usually protected by fibrous tissue, but the proximity of the distal end of the AV node is obviously an issue, and an accelerating or rapid junctional rhythm during radiofrequency application is a warning sign to stop immediately. Finally, since this issue of risk-benefit is crucial for an informed mutual decision, for the patient with asymptomatic or not yet symptomatic WPW, it would be optimal to consult a center with a significant case load of WPW patients.

We would argue that the major concern is to prevent SCD rather than avoid an ablation, and ablation of an accessory pathway is presently the only therapy that can abolish such a risk factor and at low complication risk. Although the a priori risk for SCD is estimated to be 0.13%/year in patients with asymptomatic pre-excitation, for the nearest 20 years ahead, the risk might exceed the risk for ablation-related severe complications. In children with WPW syndrome (those with congenital heart disease or cardiomyopathy excluded), the risk for VF and/or cardiac arrest was 1.9/1000 person-years (0.19%/year) and 70 times higher than in controls.^8^

Second, during the last decades there has been a debate about the role and cost-benefit of including ECG as part of preparticipation health screening in an attempt to prevent cardiac arrest and SCD during sports activities, but also in healthy young adults in general. Since 2010 reports have appeared stating that ECG is unnecessary while others offer the opposite opinion, advocating its inclusion and arguing that it also might be cost effective.^4^^,^^9^, ^10^, ^11^, ^12^ In 2020, at the time when this young man was referred to the authors, the title of an editorial commentary by Whitehill and Campbell in the October issue of the *Heart Rhythm* journal was “The ‘Great Debate’ continues,”^13^ alluding to an original article in the same issue^14^ and implying that whether to include ECG in preparticipation screening was not settled. It is outside the space and scope for this case report to discuss all pros and cons. It is, however, clear that including ECG in preparticipation screening increases the sensitivity of detecting cardiovascular abnormalities associated with SCD up to 6 times, including in persons with asymptomatic pre-excitation/WPW,^12^^,^^14^ and that the cost is reasonable^10^^,^^11^ but the price is a proportion of false-positives that must be dealt with.^12^^,^^14^^,^^15^

Finally, the postablation ECG shows signs of activation-induced cardiac memory that might be misinterpreted as due to myocardial ischemia.^5^ In this case ischemia might be suspected in the anteroseptal region of the heart supplied by the left anterior descending artery far from the left lateral ablation site, which is closer to the left circumflex artery (Figure 3).

## Conclusion

With a 3-year perspective, it can be concluded that this patient was cured from a condition that presented a threat not only to his lifestyle and future career but also to his life.

## Disclosures

The authors have nothing to disclose.
